# Nonadditive Entropy Application to Detrended Force Sensor Data to Indicate Balance Disorder of Patients with Vestibular System Dysfunction

**DOI:** 10.3390/e25101385

**Published:** 2023-09-27

**Authors:** Harun Yaşar Köse, Serhat İkizoğlu

**Affiliations:** 1 Department of Mechatronics Engineering, Faculty of Electric and Electronics, Istanbul Technical University (ITU), 34469 Istanbul, Türkiye; kose21@itu.edu.tr; 2 Department of Control and Automation Engineering, Faculty of Electric and Electronics, Istanbul Technical University (ITU), 34469 Istanbul, Türkiye

**Keywords:** vestibular disorders, insole force sensors, gait analysis, Tsallis entropy, detrending, feature extraction, classification

## Abstract

The healthy function of the vestibular system (VS) is of vital importance for individuals to carry out their daily activities independently and safely. This study carries out Tsallis entropy (TE)-based analysis on insole force sensor data in order to extract features to differentiate between healthy and VS-diseased individuals. Using a specifically developed algorithm, we detrend the acquired data to examine the fluctuation around the trend curve in order to consider the individual’s walking habit and thus increase the accuracy in diagnosis. It is observed that the TE value increases for diseased people as an indicator of the problem of maintaining balance. As one of the main contributions of this study, in contrast to studies in the literature that focus on gait dynamics requiring extensive walking time, we directly process the instantaneous pressure values, enabling a significant reduction in the data acquisition period. The extracted feature set is then inputted into fundamental classification algorithms, with support vector machine (SVM) demonstrating the highest performance, achieving an average accuracy of 95%. This study constitutes a significant step in a larger project aiming to identify the specific VS disease together with its stage. The performance achieved in this study provides a strong motivation to further explore this topic.

## 1. Introduction

The vestibular system (VS) is a perceptual system responsible for providing the brain with information regarding spatial orientation, head position, and motion. Additionally, it plays a crucial role in maintaining balance and stability [1]. Despite numerous studies in various medical fields, the detection of vestibular disorders is an area that has not received sufficient attention yet. This study aims to fill this gap by utilizing Tsallis entropy (TE) as a tool to identify VS-related diseases.

Various methods are employed in the literature to identify the specific VS problem but the most popular clinical method is still computerized dynamic posturography (CDP) [2]. The state-of-the-art methods are based on utilizing classification techniques following a machine learning step where the features are extracted from gait data. Gait data refer to the collection of information about an individual’s walking patterns and habits. They capture various aspects of walking, such as force, rhythm, speed, and variability in different components of the gait cycle. The gait cycle is a complex activity consisting of two main phases: the stance phase, in which the foot remains on the ground, and the swing phase, in which the foot moves forward. By analyzing gait data, we can detect irregularities and deviations that differ from what is considered a ‘normal’ gait. These deviations can be indicative of a variety of health conditions, from musculoskeletal problems to neurological disorders.

The gait data are especially used to give information about balance disorders related to different diseases. Within this context, gait analysis has emerged as a valuable tool in the diagnosis and monitoring of neurodegenerative diseases, providing objective measures to assess motor impairments associated with these conditions. It has been extensively utilized in the evaluation of diseases such as Parkinson’s disease (PD), Huntington’s disease (HD), amyotrophic lateral sclerosis (ALS), and other related disorders. Numerous studies have demonstrated the effectiveness of gait analysis in identifying disease-specific gait abnormalities and distinguishing between different neurodegenerative conditions. As an example, Nir Giladi et al. proposed a new clinical classification scheme for gait and posture and discussed the use of gait analysis in identifying disease-specific gait abnormalities [3]. Bovonsunthonchai et al. investigated the use of spatiotemporal gait variables in distinguishing between three cognitive status groups and discussed the potential of gait analysis as a tool for early detection of neurodegenerative conditions [4]. Guo Yao et al. summarized the research on the effectiveness and accuracy of different gait analysis systems and machine learning algorithms in detecting Parkinson’s disease based on gait analysis [5]. 

As an example of the use of gait data to evaluate balance disorders associated with dysfunction in the VS, A. R. Wagner et al. discussed how gait analysis can be used to assess vestibular-related impairments in older adults, and how these impairments can impact balance control [6]. In [7], Ikizoğlu and Heyderov search for significant features from IMU-sensor-based data to diagnose VS disorders. In [8], Agrawal et al. utilize wireless pressure sensors embedded in insoles along with machine learning models to predict fall risks, achieving promising results. In [9], Schmidheiny et al. focus on the discriminant validity and test–retest reproducibility of a gait assessment in patients with vestibular dysfunction.

In this study, our aim was to utilize contemporary classification methods to extract pertinent characteristics from gait data for the purpose of diagnosing VS-dysfunction-based balance disorders. To accomplish this objective, we employed an innovative approach that involved TE values as the feature. TE offers a framework for characterizing the statistical properties of complex systems and thus it is capable of defining non-extensive systems. TE has proven to be effective in diverse domains such as physics, information theory, and economics, enabling a more comprehensive analysis and understanding of systems with long-range correlations and heavy-tailed distributions [10]. As an example of the application of TE in the field of biomedical engineering, Zhang et al. investigated the dependency of the TE of EEG data on the burst signals after cardiac arrest [11]. Similarly, Tong et al. used the TE of EEG signals as a measure of brain injury in their study [12]. Considering the human gait to exhibit non-extensive behavior with long range correlations [13,14,15,16], we expected TE to be rather helpful in analyzing the balance performance of individuals. Thus, by applying TE to gait data, our objective was to capture vital information concerning the behavior and dynamics of the VS, which can contribute to the identification of related diseases. 

This study is an important step within a larger project which we are conducting together with the audiologists at The Medical School Cerrahpaşa-Istanbul. We aim to develop a diagnosing system to identify the specific disease that is the source of the VS dysfunction causing imbalance. We also aim to determine the stage of the problem. The first step in this process is the classification of the individual as healthy or suffering. For this classification, we are searching for primary discriminative features. We collect various features which will then enter a feature reduction/selection process. According to the experience of the audiologists, these primary features are expected to be obtained from relatively short data acquisition periods, in order to not put the patient in stress, and thus increase the accuracy of the whole system. In [7], we discussed the effectiveness of features obtained from IMU sensor data, such as average step length, average speed, step symmetry, knee bending angle, lateral/posterior waist swing, etc., where we achieved an accuracy around 90%. In [17], we presented a feature based on insole pressure sensor data called fractal spectrum width that had an accuracy around 98% in distinguishing between the classes in the first step of the entire process. This study is also based on the same data as that one, but it looks for new features based on Tsallis entropy that would be effective in the feature selection/reduction process. We set our accuracy threshold as 90% for any individual feature to advance to the reduction stage. 

We can briefly summarize the contributions we have brought with this study as follows: Most studies have focused on features related to gait analysis, such as stride time, stance time, etc., which require a relatively long walking time. This study aims to shorten the data acquisition period by capturing features from short walks. Pressure data collected from wearable insole sensors are used for feature extraction. This approach allows data to be obtained in daily life, helping the patient avoid the stress of the clinical environment and potentially improving the accuracy of the diagnosis [18,19]. We detrend the normalized raw data, allowing the identification of individual specific fluctuations around the trend, thereby increasing the accuracy. As one of our basic contributions, we propose a specific algorithm to determine the trend curve in each walking step. This process leads to a better ability to distinguish temporary imbalance from unusual walking habits. 

After feature extraction, the extracted features were used to train models using classification methods. The main classification categories included decision trees (DT), discriminant analysis, logistic regression, naïve Bayes, support vector machine (SVM), k-nearest neighbors (KNN), kernel approximation, ensemble, and neural networks. 

Considering the flow of the study, the rest of this article is structured as follows: The Materials and Methods section provides comprehensive details on TE. Subsequently, in the Data Acquisition Process section, a thorough explanation is given regarding the data collection process. In the Data Processing section, the step-by-step procedures for transforming the raw data into distinct features are elaborated upon. The outcomes of the subsequent experiments are presented comparatively within the Results section. Lastly, in the Discussion section, the results are analyzed, inferences are drawn, and future prospects regarding the utilization of the outcomes within the broader project are mentioned.

## 2. Materials and Methods

### 2.1. Entropy, Tsallis Entropy—Brief Background

Entropy is a property that is mostly used as a measure to describe the chaotic level of a dynamic system. The well-known Shannon entropy (SE) based on Boltzmann–Gibbs statistical mechanics and formulated as

(1) $$SE=-\sum_{i=1}^{N}p_{i}ln{(p}_{i}),$$

is capable of describing the structure of extensive systems with short-term microscopic correlations [20,21]. In (1), the Boltzmann constant is taken as k=1, *N* is the number of microstates, and $p_{i}$ stands for the probability of the i-th microstate.

For systems with long-term interactions, however, or systems presenting long-term memory effect, the effectiveness of applying SE for the abovementioned purpose decreases [22]. At this point, forming the generalized structure of Boltzmann–Gibbs statistics, the Tsallis entropy (TE) within the non-extensive statistics contributes significantly to finding the hidden information in the time series [23].

TE has found applications in various fields, including biomedical research. In the context of biomedicine, TE has proven to be a valuable tool for analyzing complex systems and understanding the dynamics of biological processes, with its main advantage being the ability to capture the non-linear and long-range dependencies present in biological systems [12,17]. 

The Tsallis entropy with k=1 is defined as

(2) $$TE=\frac{1}{q-1}\left(1-\sum_{i=1}^{N}p_{i}^{q}\right),$$

where q (qϵR) is a parameter to indicate the degree of non-additivity [24]. This is because, for two independent systems X and Y, we have

(3) $$TE\left(X+Y\right)=TE\left(X\right)+TE\left(Y\right)+\left(1-q\right)TE\left(X\right)TE(Y),$$

where (1 − q) is a measure of deviation from additivity. q>1 and q<1 correspond to sub-extensive and super-extensive statistics, respectively [12,25]. For q=1 we have TE = SE, corresponding to extensive statistics. In (2), N is the number of possible states and $p_{i}$ represents the probability of the i-th state. The determination of the value of the parameter q does not have specific criteria, but rather depends on the specific characteristics of the analyzed dataset [26]. By adjusting the value of q, the entropy metric can be tailored to capture particular features inherent in the analyzed dataset.

### 2.2. Data Collection

We recall that the data used in this study are the same as in our previous study [17].

**Figure 1:**
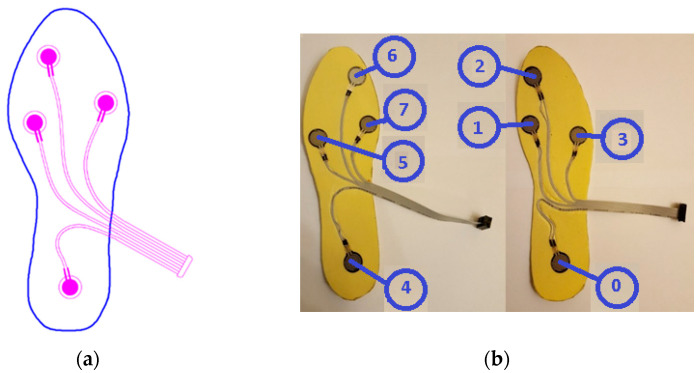
(**a**) Sensor placement on the insole; (**b**) numbering of the sensors S0 to S7 (top view) [17].

When the gait analysis studies in the literature are examined, it is seen that the distribution of weight is concentrated especially at four main points on the soles of the feet, as depicted in Figure 1a [27,28,29,30,31]. Also in this study, these four points were chosen for the placement of the sensors in line with the opinions of several academics in the field of audiology, who are acknowledged in the Acknowledgments section.

To ensure data collection without disturbing the natural walking patterns of the participants, 5 pairs of insoles with different sizes (36, 38, 40, 42, 44—according to European standards) were manufactured. Prior to the commencement of the experiment, the correctly sized insoles were inserted into the subjects’ shoes. For the production of the insoles, a durable and soft plastic material commonly employed in the manufacturing of orthopedic products was utilized.

Force-sensitive resistors (FSR) were chosen as pressure sensors, as they are widely used in gait analysis applications and offer several advantages [32]. Considering the physical dimensions and the acceptable repeatability feature, the FSR402-short tail model from Interlink was selected [33]. The characteristics of the sensor can be found in Table 1. The sensors on the insoles were numbered S0 to S7, as seen in Figure 1b.

Some explanatory information about the characteristics in Table 1 can be given as follows: Repeatability is a measure of the scattering of results for multiple measurements under the same conditions. For our sensor, the maximum deviation of the results of successive measurements of the same measurand from the mean is given as ±2%. Idle resistance is the resistance of the resistive force sensor when no force is applied to it. Hysteresis is a measure of how far the system output is different depending on whether a specific input value was reached by increasing vs. decreasing the input. Rise time is the time it takes for the system/sensor output to change from 10% to 90% of its final value. This time, given in Table 1 as less than 3 microseconds, shows that the sensor responds rapidly to the force/change in force applied to it.

Data collection was carried out in the clinical setting of the Audiology Department at Cerrahpaşa Medical School, Istanbul University—Istanbul, Türkiye. The process was conducted in compliance with the principles outlined in the Helsinki Declaration. Before starting the process, approval was obtained from the Istanbul University Ethics Committee (Approval number: A-57/07.07.2015). In addition, informed consent was obtained from all subjects before participation in the study. For individuals with VS problems, their conditions had already been diagnosed by the audiologists using conventional systems (computerized dynamic posturography-CDP).

Data were collected on weekends to minimize the subjects’ stress and avoid interference from other nearby devices. The subjects were asked to walk the 12 m long path twice. The first walk aimed to help them become familiar with the environment and reduce any possible stress, while the data from the second walk were used for analysis in general. In some cases, subjects walked a third time when needed as a result of the audiologists’ observations. 

The pressure sensor data collected with the Arduino Mega device placed on the subjects were transferred to the laptop wirelessly via an HC-06 Bluetooth unit. Sampling was performed from all sensors simultaneously at a rate of 20 samples per second. In order to convert the force to voltage, a 1 kΩ resistor in series with the FSR served as a voltage divider. As the next step, we calibrated this structure in the lab since the FSR has a highly non-linear characteristic curve. Supplying the structure with 5 V DC voltage presented an average function as

(4) $$w=e^{\frac{v_{o}+0.2245}{0.9265}},$$

where w (N) is the weight applied onto the sensor and $v_{o}$ (V) is the output voltage. A 10% deviation from the values obtained by Equation (4) was taken as the criterion that would disqualify the relevant sensor from being used in the experiments.

Informative data about the participants are listed in Table 2.

The distribution of the subjects whose specific disease was detected by CDP by audiologists is given in Table 3.

To ensure the confidentiality and privacy of all participants, their identities have been anonymized for publication of this article.

### 2.3. Data Processing

In order to interpret the results more accurately on the basis of the subject, the obtained data were preprocessed before feature extraction. Thus, the feature extraction process was carried out in six stages.

Stage 1—Framing useful data

We framed the useful part of the whole walk, and data corresponding to the first and last steps were extracted from the overall data. Thus, data on steps with missing dynamic behavior were excluded from the evaluation.

Stage 2—Determining the intervals when the foot is actively touching the ground

Of all the gait data, only those corresponding to the time intervals during which the foot is actively touching the ground provide useful information. These intervals were determined for each foot as follows:

- All the sensor data were normalized to the range 0–1 as

(5) $$X_{norm}=\frac{X-X_{min}}{X_{max}-X_{min}}\;,$$

where X is the original/raw data and $X_{min}\;$ and $X_{max}$ represent the minimum and maximum values, respectively.

- The maximum of all sensor data ($S_{max}$) was determined. As an example, for the right foot, these data were obtained as $S_{Rmax}=\max\left(S_{0},\;S_{1},\;S_{2},\;S_{3}\right)$.
- A threshold was set so that the foot was interpreted as being in the air for the time interval where $S_{max}$ remained below this threshold value.

**Figure 2:**
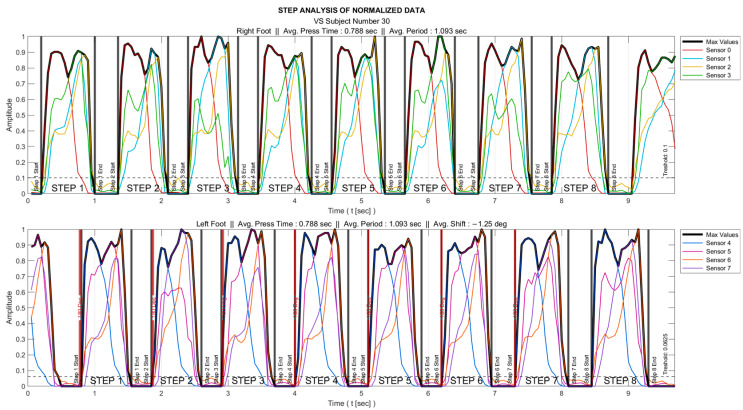
Normalizing the data followed by determining the intervals when the foot is actively touching the ground.

The process is visualized for a sample subject in Figure 2; there, the individual sensor data are marked in different colors, their maximum in black, and the foot-in-the-air position is shown as zero amplitude.

Stage 3—Interpolation

**Figure 3:**
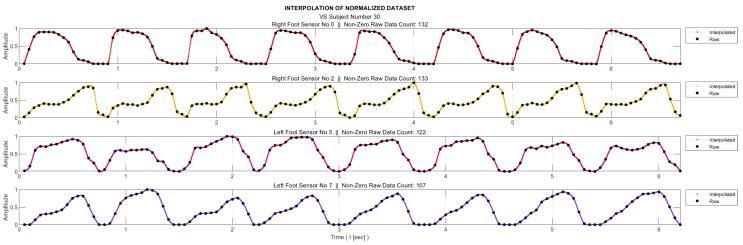
Twenty-fold interpolated data of some sensors after removal of segments where the foot does not actively touch the floor.

As mentioned in the ‘Data Collection’ section, the sampling frequency for data acquisition was 20 Hz. On the other hand, for meaningful entropy calculation, we need a significant number of bins in the histogram of the relevant data, as well as a sufficient number of samples in each bin. Therefore, we applied 20-fold interpolation to all the sensor data. Prior to the interpolation process, the segments where the feet were not in contact with the ground were removed from the data sequences. The process is illustrated in Figure 3 for a sample subject. Linear interpolation was not preferred in order to maintain accuracy without compromising the representation of the data. Instead, the cubic Hermite interpolation method was chosen as the interpolation technique. This method provides a smoother and more accurate representation of the data while preserving its integrity [34].

Stage 4—Detrending

**Figure 4:**
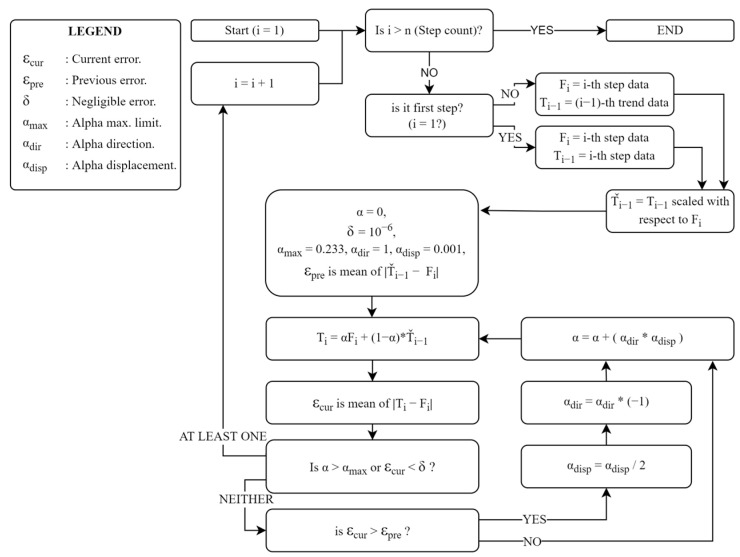
Flowchart of the algorithm developed to generate the stepwise trend curves.

To classify an individual as healthy or diseased, we are concerned with the deviation of the data from those corresponding to the person’s walking habit. Therefore, we first determined the trend data related to the walking habit. The process illustrated in Figure 4 can be briefly explained as follows: For each step, the trend curve of the previous step is scaled in the time axis using the ‘nearest-neighbor interpolation’ method based on the length of the current step data; thus, we equate both the current and previous step data lengths. A trend dataset is then generated for the current step i using Equation (6).

(6) $$\begin{array}{c} T_{i}=F_{i}\;\;\;\;\;\;\;\;\;\;\;\;\;\;\;\;\;\;\;\;\;\;for\;\;\;\;\;\;\;\;\;\;\;\;i=1\\ T_{i}=\alpha F_{i}+\left(1-\alpha \right){\overset{ˇ}{T}}_{i-1}\;\;\;\;\;\;\;\;for\;\;\;\;\;\;\;\;\;\;\;\;i=2,\;3,\ldots ,\;\;n. \end{array}$$

Here, $T_{i}$ is the current-step trend data, and $F_{i}$ stands for the current step data. $\overset{ˇ}{T}$ denotes the trend data whose length is scaled, and α is a coefficient indicating the degree to which the previous trend curve is approximated to the current step data set. $\alpha_{max}$ represents the maximum rate of change that each data point of the trend curve can exhibit from one step to the next, for which the value 0.23 was statistically determined, considering data from healthy subjects. We note that $\alpha_{max}$ serves as a parameter to achieve a balance between flexibility in trend curve adaptation and avoiding overfitting, and although it has a role in shaping the trend curve, the key features of our analysis remain relatively insensitive to its exact value. The process is terminated when the α value reaches $\alpha_{max}$ or the error defined as $\varepsilon =mean\;\left\{\left|T_{i}-F_{i}\right|\right\}$ falls below a threshold so that it is considered negligible. The threshold level is set as $10^{-6}$. 

**Figure 5:**
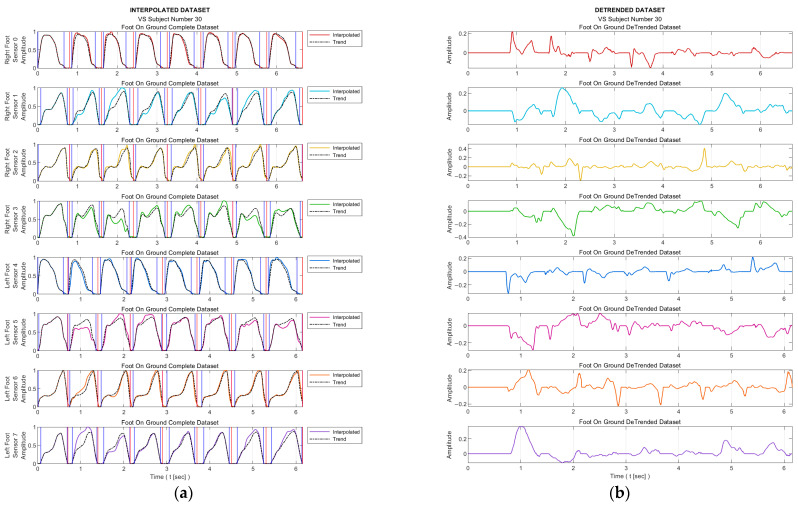
(**a**) Trend curves and (**b**) curves of detrended dataset for a sample VS-diseased subject. Red vertical lines indicate the active stepping intervals of the foot; blue vertical lines indicate the active usage intervals of the relevant sensor.

Figure 5 presents the trend curves and the detrended dataset for a sample VS-diseased subject.

Stage 5—Tsallis Entropy Calculations

**Figure 6:**
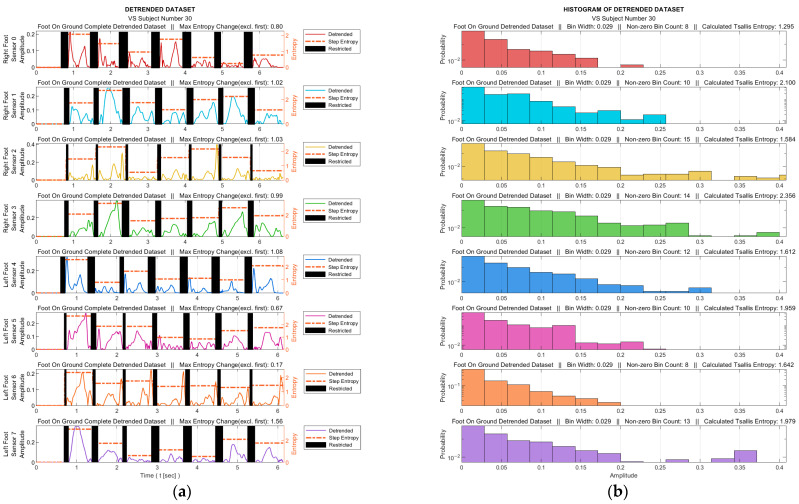
For a sample diseased subject (no. 30): (**a**) absolute values of the detrended data in Figure 5b and the step-by-step TE values (black bars indicate ranges in which the corresponding sensor is inactive); (**b**) histograms derived from the data for the entire gait (sensor-inactive intervals removed).

At this stage, the TE calculation was performed with the help of the histograms generated from the detrended data. The process was performed for both the data for the entire gait from each sensor and for all the step data within the gait cycle. For each sensor, the data corresponding to the intervals in which the relevant sensor was not actively used were extracted from the data set. These intervals are marked as black bars in Figure 6a for a sample data set. Histograms were obtained from the absolute values of the detrended dataset, where the maximum number of bins was determined as 25 in order to achieve an acceptable granularity. Figure 6b illustrates the corresponding histograms for the data set in Figure 6a.

**Figure 7:**
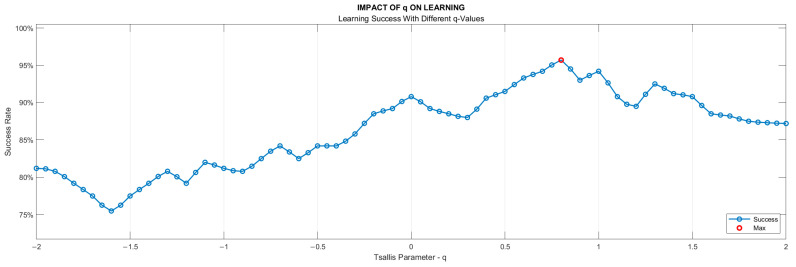
Dependency of the learning success on Tsallis parameter (q) value.

As mentioned in Section 2.1, the selection of the q parameter value in TE calculation does not have a predefined criterion, it rather depends on the specific characteristics of the analyzed data set. The best q value that would achieve the highest accuracy for our data sets and therefore maximize the discriminatory power of TE was determined to be 0.82 by an iterative process. In the process of determining the q value, nine classification algorithms of the learning models outlined in Stage 6 took part with a 10-fold cross-validation technique. The ratios of the models attaining the highest success were employed as the benchmark. The learning success rates vs. q values are depicted in Figure 7.

Stage 6—Feature Extraction

As stated in the introduction, although human gait seems to have a regular pattern, a literature review reveals that fluctuations are observed in this pattern. For healthy people, these fluctuations are long-range correlated. However, this correlation weakens for people with balance problems. Thus, the TE value could be a significant measure to classify individuals as healthy or diseased. In this study, we leveraged two TE-based possibilities to identify VS-dysfunction-based problems. One was to consider the TE value of the entire gait cycle, and the other was to examine the change in TE value from step to step. For the second case, we decided to examine the deviation of the TE value from zero, because in the ideal case it is clear that the step-to-step change of entropy for a healthy person would be zero. Thus, for this case, the data set containing the step-by-step entropy values was expanded by adding the negatives of all data values, and the standard deviation of the newly created data set ($\sigma \left(E^{\prime }\right)$) was calculated as given by Equation (7).

(7) $$\begin{array}{c} E=\left\{e_{1},e_{2},\ldots ,e_{n}\right\}\;\;where\;e_{k}\in Rfor\;\;k\in Z^{+},\\ E^{\prime }=\left\{e_{1},e_{2},\ldots ,e_{n},{-e}_{1},{-e}_{2},\ldots ,{-e}_{n}\right\}=\left\{x_{1},x_{2},\ldots ,x_{n},x_{n+1},x_{n+2},\ldots ,x_{2n}\right\}\\ \sigma \left(E^{\prime }\right)=\sqrt{\frac{1}{2n}*\sum_{i=1}^{2n}{\left(x_{i}-\mu \right)}^{2}} \end{array}$$

In Equation (7), $e_{k}$ is the TE value of the k-th step data, E denotes the set of step-by-step TE values, and $E^{\prime }$ represents the expanded set.

We had four sensors under each foot, so, eight sensors in total. Using both the TE value of the entire gait cycle for each sensor as well as the stepwise variation in the TEs, we had a total of 16 features that served for machine learning. For the classification process, we used the Matlab R2021b Classification Learner Tool (on MSI GE75 Raider 10875H). A 10-fold cross-validation technique was applied, where approximately 25% of the total data (from 15 subjects) was used for testing and the remainder (from 45 subjects) for training.

The process of classification training involved utilizing nine different model categories: decision trees (DT), discriminant analysis, logistic regression, naïve Bayes, support vector machine (SVM), k-nearest neighbors (KNN), kernel approximation, ensemble, and neural networks. Considering the sub-models of these categories that were used, such as ‘Course: 4, Medium: 20, Fine: 100’ for the maximum number of splits in the decision tree category, a total of thirty-two models were involved in the process. 

Among all the classifiers examined, SVM (Gaussian), KNN (cosine, k = 10), and logistic regression showed the three best performances. Regarding these classifiers, the KNN algorithm determines the class membership of an object/vector by examining its k nearest neighbors [35]. In this study, the k value yielding the best result was determined to be 10. Logistic regression is a statistical model used to predict the probability of a dependent variable belonging to two or more classes in a dataset [36]. SVM seeks to find an optimal hyperplane to separate data clusters [37]. These three algorithms are among the most widely used in studies on biomedical signals in the literature [38,39,40,41,42,43].

## 3. Results

In this section, a comparative analysis is made based on data collected from both healthy and VS-diseased individuals. The comparison commences from the detrending stage of processing the sensor data, as described in the Data Processing section.

**Figure 8:**
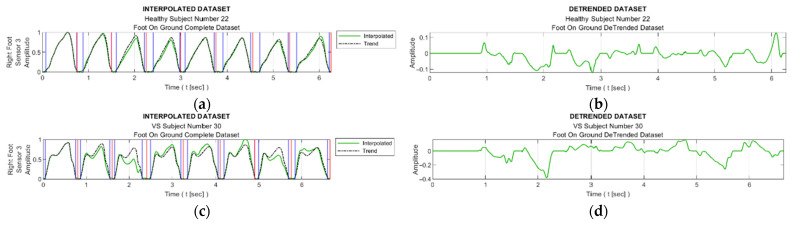
Sample interpolated S3 sensory data and the stepwise trend curves of (**a**) a healthy subject and (**c**) a VS-diseased subject; detrended data from (**b**) a healthy subject and (**d**) a VS-diseased subject.

Figure 8 facilitates observation of discernible variations in the data from sensor S3 during walking for sample healthy and diseased individuals. Additionally, it visualizes the detrended data, i.e., the difference between the step data and the trend curve.

To see the effect of the proposed trending algorithm, trend curves were created using 2nd-, 3rd-, and 4th-degree curve-fitting polynomials and the results were compared. The classification accuracies obtained with the different trending methods are listed in Table 4.

**Figure 9:**
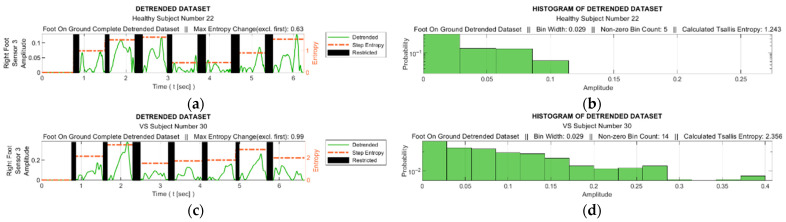
(**a**,**c**) Detrended data with absolute values taken from Figure 8b,d; (**b**,**d**) histograms produced from these graphs.

**Figure 10:**
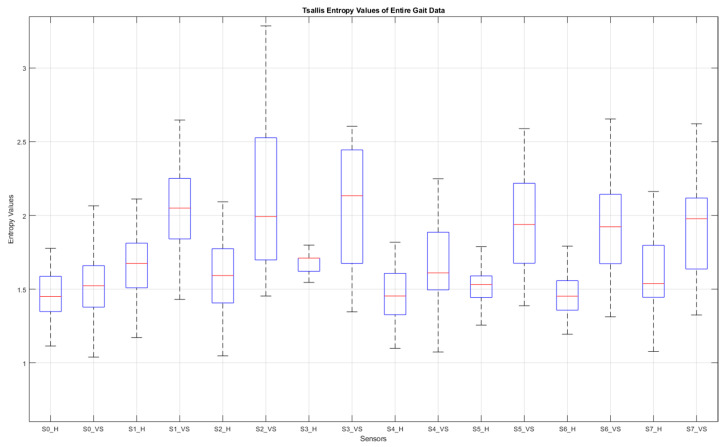
Box plot of the entire-gait TE values for all participants. S: sensor, H: healthy, VS: diseased.

Figure 9 shows graphs of the detrended data with absolute values taken from Figure 8b,d and the histograms produced from these graphs. In Figure 9a,c, the black bars indicate the inactive periods of the related sensor. For these sample subjects and sensor data, the maximum step-by-step change in the TE value for the healthy subject was calculated as 0.63, whereas it was 0.99 for the VS-diseased person. The TE value for the entire gait cycle was calculated as 1.243 for the healthy individual and 2.356 for the suffering subject. In Table 5, the TE values are listed for these sample subjects for all sensor data. Figure 10 summarizes the entire-gait TE values for all participants.

**Figure 11:**
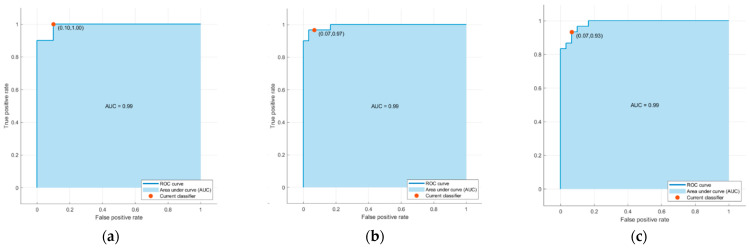
ROC curves associated with (**a**) the support vector machine (SVM) model with Gaussian kernel, (**b**) logistic regression, and (**c**) the k-nearest neighbors (KNN) algorithm using cosine similarity in Table 7.

As described in Data Processing section, thirty-two classifiers provided by the Classification Learner Tool in Matlab were trained using sixteen features with ten-fold cross-validation. The average accuracies of the major classification algorithms are listed in Table 6 and Table 7 and Figure 11 display the confusion matrices and corresponding receiver operating characteristic (ROC) curves for one of the ten training test set pairs of the top three classifiers.

## 4. Discussion

This study was carried out in conjunction with a project where our ultimate goal is to identify the specific diseases of individuals suffering from VS dysfunction, along with the level of the problem. In the full version of the project, a machine learning process will be conducted using distinctive features as input. For this purpose, features that will be effective in defining the problem are being sought and all of them will be placed in the candidate features basket, that is, they will be selected to take part in the feature reduction stage. According to the experience of the audiologists with whom we conducted the experiments, some important points need to be considered when collecting data from patients in order to achieve a high level of accuracy in diagnosis. These are particularly obtaining the data in a short time and collecting it under stress-free conditions. Having taken these guidelines into account, and thus aiming to capture the features from a short walk, we performed multifractal detrended fluctuation analysis (MFDFA) in our previous study [17]. Our current study also used these same data as our previous work but it provided additional features for the feature selection/reduction step. 

In this study, we utilized TE-based methods for feature extraction from gait data collected from insole pressure/force sensors. The reason for considering the TE was its ability to capture the level of the fluctuations in the detrended data, providing insight into the complexity and irregularity of the gait pattern. Unlike other entropies, TE enables a parameterized analysis, offering flexibility in quantifying uncertainty and capturing certain characteristics of the data distribution.

Data from eight insole sensors, four under each foot, were first normalized and then detrended to provide information about fluctuation around the trend curve of the individual. With this process, we aimed to consider the gait habit of the person in order not to misinterpret an unusual gait habit as identifying a balance disorder. As one of the effective innovations brought by this study, we developed an algorithm that determines the trend curve at each step. The efficiency of this algorithm can be seen when the results are compared with other curve fitting methods. Using our algorithm, we achieved an average accuracy of 95% in distinguishing VS patients from healthy subjects, while the best rate was 86.7% even with a fourth-order curve-fitting polynomial. A total of sixteen features were involved in the classification process, eight of which were derived from the TEs of the entire gait cycle and the other eight from the step-by-step TE change for each sensor. The TE value for the entire gait cycle and the step-by-step variation in the TE value were observed to be greater in VS patients than in healthy individuals, which we explained by the high data deviation around the trend curve for these individuals. The TE parameter q was determined experimentally as 0.82. As we can see from Figure 10, of all the sensor data, those from the under-the-heel sensors (S0 and S4) contributed the least to the classification process, such that the differences in TE values for these data were the smallest. This is easy to explain, as the sensors in question were placed at points where even a diseased person does not show a significant fluctuation.

Regarding the data collection time, the subjects had to walk for around 10–15 s. As we described in detail in [17], this time period is much shorter than most experiments in the literature, meeting the expectations of the respected audiologists we consulted with throughout the project. Despite such a short test time, high accuracy was achieved by processing the instantaneous values of the gait data using appropriate methods rather than dealing with step-based features such as stride time, stance time, etc.

The SVM with Gaussian kernel and logistic regression performed best in the classification process with 95%, followed by KNN (cosine) and neural network (wide) with 93.3%. At this point, we would like to emphasize that we had defined our criterion for categorizing any feature as distinctive and labeling it as a candidate for feature reduction as an individual accuracy level threshold of 90% [17]; thus, the TE-based features passed this evaluation stage successfully. On the other hand, we believe that a more reliable result will be achieved with an increase in the number of participants.

In addition to the numerical values presented in the Results section, we provide further statistical data in Table 8, in order to provide a fuller picture of the results.

Currently, we are conducting experiments for the binary classification phase of the larger project so that an individual can be described as ‘suffering’ or ‘healthy’. As we stated in [17], features that take into account the trends specific to an individual are expected to be quite effective in determining the stage of the problem. So, we look forward to using these features also for this future step of the whole project.

## Figures and Tables

**Table 1:** Characteristics of the FSR402-short tail sensors.

Parameter	Value
operation range	0.2 N–20 N
physical dimensions	ϕ_pad_ 18.3 mm, ϕ_sens_ 12.7 mm
thickness	0.46 mm
repeatability	±2%
idle resistance	>10 MΩ
hysteresis	10% max.
rising time	<3 µs

**Table 2:** Information about the subjects.

	Healthy (30)		Diseased (30)	
	Male (15)	Female (15)	Male (13)	Female (17)
age	54.3 ± 8.5	55.1 ± 7.9	54.5 ± 8.5	56.8 ± 7.2
mass (kg)	66.6 ± 9.8	65.1 ± 8.8	65.9 ± 10.2	64.9 ± 7.9
height (cm)	169.2 ± 10.0	164.0 ± 6.2	170.3 ± 8.8	163.4 ± 5.7

**Table 3:** The distribution of diseased subjects.

	Male	Female
BPPV *	6	8
UVW *	3	4
Meniere	3	3
Vestibular Neuritis	1	2

(*) BPPV—benign paroxysmal positional vertigo, UVW—unilateral vestibular weakness.

**Table 4:** Classification accuracies with different trend generation methods.

Classification Model	Proposed Algorithm	Second-Degree Polynomial	Third-Degree Polynomial	Fourth-Degree Polynomial
SVM-Gaussian	95.0%	71.7%	76.3%	81.7%
Logistic regression (LR)	95.0%	63.3%	78.3%	76.3%
KNN-cosine	93.3%	66.7%	70.0%	78.3%
Model with highest accuracy	95.0% (with SVM-G and LR)	83.3% (with Ensemble-Bagged Trees)	83.3% (with Decision Trees-Fine/Med.)	86.7% (with Ensemble Subsp. Discr.)

**Table 5:** TE values calculated from each sensor’s data for sample subjects.

	Healthy Subject (no. 22)		VS Subject (no. 30)	
Sensor	Entire Gait	Stepwise Max	Entire Gait	Stepwise Max
S0	1.39	0.98	1.29	0.80
S1	2.15	0.83	2.10	1.02
S2	1.38	0.72	1.58	1.03
S3	1.24	0.63	2.36	0.99
S4	1.08	0.87	1.61	1.08
S5	1.38	0.79	1.96	0.67
S6	1.36	0.82	1.64	0.17
S7	1.54	0.86	1.98	1.56

**Table 6:** Accuracy of major classification algorithms.

Algorithm	Accuracy (%)
SVM (Gaussian)	95.0
Logistic regression	95.0
KNN (cosine)	93.3
Neural network (wide)	93.3
Kernel (SVM)	91.7
Ensemble (bagged tree)	88.3
Naïve Bayes (kernel)	86.7
Quadratic discriminant	78.3
Decision tree (fine)	73.3

**Table 7:** Confusion matrices for one of the ten training test set pairs.

Predicted Class	SVM (Gaussian)		Logistic Regression		KNN (Cosine)	
	H	D	H	D	H	D
H	30	0	29	1	28	2
D	3	27	2	27	2	28

**Table 8:** Some statistical data about the top two classification algorithms.

Statistical Property	SVM (Gaussian)	Logistic Regression
accuracy (%)	95.0	95.0
sensitivity (%)	91.6	94.0
specificity (%)	97.9	95.1
F1 Score	0.945	0.943
MCC	0.899	0.891

## Data Availability

The data are not publicly available due to confidentiality agreements and privacy concerns of the participants, as stated in the consent form.
